# Casual Sexual Scripts on the Screen: A Quantitative Content Analysis

**DOI:** 10.1007/s10508-018-1147-1

**Published:** 2018-03-27

**Authors:** Elisabeth Timmermans, Jan Van den Bulck

**Affiliations:** 1 Department of Media and Communication, Erasmus School of History, Culture and Communication, Erasmus University Rotterdam, 3062 PA Rotterdam, The Netherlands; 2 Department of Communication Studies, University of Michigan, Ann Arbor, MI USA

**Keywords:** Casual sex, Content analysis, Television, Sexual script, Sexual behavior

## Abstract

While existing content analyses have provided insightful information in terms of contextual factors and frequency of sexual behaviors, not much is known about the relational context in which sexual depictions generally occur. The current study addresses this void by employing content analytic methods to measure the frequency and context of depictions of sexual behavior within nine popular television shows produced in the U.S., while taking into account the type of sexual behavior. The results suggest that, in the analyzed television shows, sexual behaviors within a casual sexual context were almost as frequently shown as sexual behaviors within a committed relationship context. Whereas sexual behaviors within a committed relationship context were mainly limited to passionate kissing, sexual behaviors within a casual sexual context mostly consisted of explicit portrayals of sexual intercourse. Additionally, genre seemed to be an important factor when examining casual sexual television content. The situational comedy genre, for example, had no explicit portrayals of intercourse and mainly portrayed kissing couples within a committed relationship. The comedy drama genre, on the contrary, had the largest proportion of explicit sexual portrayals, usually between casual sexual partners. A second goal of this study was to analyze the portrayals of the typical casual sexual experience script and the typical casual sexual relationship script in which these sexual behaviors often occur. For instance, our analyses revealed that female characters were more likely to initiate casual sex compared to male characters. Moreover, casual sex often occurred between former partners.

## Introduction

Recently, an expanding body of literature has investigated the prevalence and consequences of casual sexual experiences and relationships (e.g., Claxton & van Dulmen, 2013; Eisenberg, Ackard, Resnick, & Neumark-Sztainer, 2009; Fielder & Carey, 2010). The predominant concerns about participation in casual sex revolve around negative mental outcomes (Bersamin et al., 2014) and physical danger such as contracting a sexually transmitted infection (Heldman & Wade, 2010; Manning, Giordano, & Longmore, 2006).

It has been shown that a number of sexual scripts exist and that casual sex can be highly scripted (e.g., Eaton, Rose, Interligi, Fernandez, & McHugh, 2016; Littleton, Tabernik, Canales, & Backstrom, 2009). Several researchers already hinted that television as a cultural storyteller partly aids in creating casual sexual scripts (e.g., Claxton & van Dulmen, 2013; Garcia, Reiber, Massey, & Merriwether, 2012; Heldman & Wade, 2010). Yet, relatively little is known about how these casual sexual scripts are portrayed in popular television shows. When reviewing existing content analyses related to sexual behavior on the screen (e.g., Aubrey, 2004; Kunkel, Eyal, Finnerty, Biely, & Donnerstein, 2005), we noted that these content analyses often do not focus on the relational context within which sexual behaviors can occur (i.e., within a committed relationship versus a casual sexual experience or relationship) nor do they differentiate between casual sexual scripts.

Accordingly, the purpose of this study was twofold. First, this study will map frequencies of sexual portrayals within its context on a number of popular U.S. television shows. Given that females, in particular, still experience some stigma when engaging in casual sex (e.g., Conley, Ziegler, & Moors, 2013), we argue that sex within a committed relationship is still more accepted. Therefore, the occurrence of sexual behaviors within casual sexual experiences and relationships will be contrasted with the occurrence of sexual behavior within more socially accepted romantic constructs such as traditional dates and romantic relationships, while taking into account the type of sexual behavior. This will be examined for three different genres relevant to the purpose of this study. Secondly, we argue that it is important to gain insights in the casual sexual experience script and the casual sexual relationship script frequently portrayed in popular fiction, as they aid in understanding what is to be expected within casual sexual scripts.

### Casual Sexual Scripts in Contemporary Society

Sexual script theory suggests that sexuality and sexual behaviors are social processes that are determined by a set of “scripts” used to organize and interpret sexual encounters into understandable conventions in which people can predict who does what and when in a particular context (Simon & Gagnon, 1969, 1986). In contrast to theories rooted in evolutionary biology (e.g., sexual strategies theory) that influence why emerging adults engage in casual sex, sexual scripts influence how emerging adults navigate their desires in a particular sociocultural context (Garcia et al., 2012). Cultural sexual scripts are the societal norms and narratives that provide guidelines for sexual behaviors such as the number of sexual partners that is appropriate, the variety of sexual acts, motives for casual sex, and suitable emotions and feelings (e.g., Mahay, Laumann, & Michaels, 2001; Sakaluk, Todd, Milhausen, Lachowsky, & URGiS, 2014).

Over the past decade, more and more researchers across the globe noticed new cultural sexual scripts on the college campus in which casual sex either occurs (1) within a casual sexual experience such as (sexual) hookups or one night stands (e.g., Campbell, 2008; Kaspar, Buß, Rogner, & Gnambs, 2016; Paul, McManus, & Hayes, 2000) or (2) within a casual sexual relationship such as friends with benefits or fuck buddies (e.g., Mongeau, Knight, Williams, Eden, & Shaw, 2013; Wentland & Reissing, 2014). Such casual sexual experiences and relationships often have their own set of rules regarding what is expected to occur in terms of sexual (and other) interactions within this context and what is not (Atwood & Dershowitz, 1992; Simon & Gagnon, 1986). Consequently, it is important to differentiate between the casual sexual experience script and the casual sexual relationship script.

A casual sexual experience is generally described as a spontaneous sexual encounter that mostly occurs in a context where friends are present and alcohol facilitates the casual sexual interaction (e.g., Bogle, 2008; Holman & Sillars, 2012; Wade, 2017). Cultural scripts about casual sexual experiences predispose that casual sexual experiences are fun, status enhancing, a reflection of one’s sexual freedom, harmless, and without emotional commitment (e.g., Aubrey & Smith, 2013; Lyons, Manning, Longmore, & Giordano, 2014).

An ongoing series of sexual encounters between two individuals is generally referred to as a casual sexual relationship. Casual sexual relationships offer sexual partners more freedom to sexually explore each other, meaning that they are characterized by higher levels of kissing as well as intimate touching and anal sex compared to casual sexual experiences (Jonason, Li, & Richardson, 2011). Communication within a casual sexual experience is often described as nonverbal (e.g., physical flirting, eye contact, dancing; Kratzer & Aubrey, 2016). Contrarily, casual sexual relationships allow for other kinds of interactions besides the sexual, thereby being more likely to create expectations of emotional involvement (Mongeau et al., 2013). However, compared to couples in committed relationships, casual sexual partners still perform less committing acts such as talking and handholding (Jonason et al., 2011). Several studies showed that between thirty and fifty percent of emerging adults had had at least one casual sexual relationship during college, with men being more likely to report engagement in casual sexual relationships compared to women (e.g., Afifi & Faulkner, 2000; Bisson & Levine, 2009; Mongeau et al., 2013; Owen & Fincham, 2011).

Overall, casual sexual scripts are perceived as less formal than the widely recognized conventions in dating scripts. Dating typically involves a rather formal pattern in which participants know one another, or want to get to know each other, and there is the possibility for a relationship (Bradshaw, Kahn, & Saville, 2010). While it is no longer men’s exclusive responsibility to pay for dates within the heterosexual dating script, the man is still supposed to initiate the date, pick up the woman, and pay for the date expenses (Morr Serewicz & Gale, 2008), whereas the woman still holds the power to choose whether or not to help pay (Lever, Frederick, & Hertz, 2015). Such heterosexual gender roles are less clear-cut within casual sexual scripts, as both men and women can initiate casual sex (Paul & Hayes, 2002). Additionally, a date is usually arranged, whereas a casual sexual encounter is often an unplanned consequence of a social gathering (e.g., a party or festival; Bogle, 2008; Holman & Sillars, 2012).

While some studies suggest that the dating script and casual sexual scripts coexist (e.g., Brimeyer & Smith, 2012), others argue that—if a date occurs—it is most likely a consequence of an unplanned casual sexual interaction, thereby implying that the casual sexual experience script precedes the dating script (Reid, Elliott, & Webber, 2011; Wade, 2017). Not only dates, but even committed relationships are often preceded by casual sexual scripts (e.g., England, Shafer, & Fogarty, 2008; Rhoades & Stanley, 2014). Yet, these casual sexual scripts might not be the ideal way to find a romantic partner, as researchers found that the sooner relationships become sexual, the greater their odds of failure (Willoughby, Carroll, & Busby, 2014). While the possibility of a romantic relationship is often a reason to start a casual sexual relationship (e.g., Furman & Hand, 2006; Mongeau et al., 2013), only a small minority of casual sexual relationships lead to committed relationships (Bisson & Levine, 2009). Even when this happens, young adults who were in a casual sexual relationship prior to becoming exclusive reported lower relationship satisfaction when compared to young adults who were not (Owen & Fincham, 2012).

Researchers also found that emerging adults generally believe that others were having more casual sexual experiences and feel more comfortable with such casual sexual experiences than themselves (Barriger & Vélez-Blasini, 2013; Napper, Kenney, & LaBrie, 2015), which is often referred to as “pluralistic ignorance” (Lambert, Kahn, & Apple, 2003; Reiber & Garcia, 2010). The pressure to act in accordance with these false perceived norms (i.e., men’s overestimation of women’s comfort and women’s overestimation of other women’s comfort) may be leading individuals to engage in behavior with which they are uncomfortable, such as engagement in casual sex (Reiber & Garcia, 2010). More than ever, emerging adults now believe that casual sex is something they are supposed to have (Wade, 2017). Emerging adults without personal experience with casual sexual relationships do not seem to have difficulties in identifying and differentiating between variations of these casual sexual relationships (Wentland & Reissing, 2014), suggesting that casual sex has become part of the culturally accepted sexual script. Several researchers already argued that television can provide narratives that partly influence these culturally accepted casual sexual scripts (e.g., Claxton & van Dulmen, 2013; Heldman & Wade, 2010; Kaspar et al., 2016). Consequently, it might be helpful to examine how television has portrayed casual sexual behavior over the past decade to better understand these casual sexual scripts.

### Sexual Behavior on the Screen

According to social cognitive theory, which is one of the most frequently cited theories to explain the influence of sexual media on an individual’s sexual scripts, viewers learn about behavior, attitudes, and beliefs by observing one or more models (Bandura, 2001). When these observed models (i.e., characters) are often having sex within a casual sexual experience or relationship rather than a committed relationship, viewers will start to perceive casual sex as part of the normative sexual script. Therefore, it is important to gain a better idea of what is already known about sexual behavior on the screen and which research gaps still need more attention.

The majority of content analyses have been carried out to examine the frequency of sexual references and behaviors in television content produced in the U.S. (e.g., Bond & Drogos, 2014; Kunkel, Eyal, Donnerstein, Biely, & Rideout, 2007; Signorielli & Bievenour, 2015). Such studies found that talk about sex is generally shown more often than sexual behavior (Kunkel et al., 2007). Others showed that emotional and social consequences of engagement in sexual behavior far outnumbered physical consequences and that female characters are more likely to experience negative consequences of sexual behavior compared to male characters (Aubrey, 2004; Eyal & Finnerty, 2009). Moreover, Hust, Brown, and L’Engle (2008) pointed out the poor representations of sexual health messages, whereas Kim et al. (2007) examined the heterosexual script on primetime network television and found that male characters most frequently were shown as actively and aggressively pursuing sex. These studies are certainly useful in gaining a better understanding of contextual factors and gender representations related to sexual references and behavior. Yet, the large majority of these content analyses does not provide any information related to the relational context in which these sexual behaviors can occur, thereby providing relatively few information related to casual sex on the screen.

When comparing findings between content analyses on soap operas conducted in 1985, 1994, and 1996, Greenberg and Woods (1999) showed that sexual activity was most commonly portrayed or talked about as occurring between two unmarried people. Remarkably, significantly fewer portrayals or sexual references of intercourse between married couples occurred. Yet, based on the coded information, it is not clear whether unmarried intercourse refers to premarital sex between committed partners or casual sexual intercourse between strangers or people in a casual sexual relationship. Kunkel et al. (2007) also provide some limited information on the prior relationship between the characters that engaged in sexual intercourse in television programs broadcasted between 1998 and 2002. The majority of characters had an established relationship (53% in 1998, 50% in 2000, and 61% in 2002). A smaller number of characters were acquainted (28% in 1998, 25% in 2000, and 19% in 2002) and only a small number of characters were basically strangers (10% in 1998, 16% in 2000, and 7% in 2002). In another study, Kunkel et al. (2005) showed that the number of characters who have just met and have sex together increased again from 7% in 2002 to 15% of all sexual intercourse scenes in between 2004 and 2005.

Similarly, Fisher, Hill, Grube, and Gruber (2004) included relationship status in their coding system and could conclude with this information that sexual intercourse most often occurred between unmarried couples and that in more than half of those instances characters were in some type of “casual sex relationship.” Notably, however, examples of an “ongoing casual sex relationship” provided by the authors such as “an affair” or “a prostitute with a regular client” (Fisher et al., 2004, p. 535) are quite different from the casual sexual relationships described in the previous section. In addition, it is not clear whether the relationship status categories “past history of romantic involvement,” “had met before in a nonromantic context,” and “had just met” were also included in this concept of casual sexual relationship as provided by Fisher et al. (2004).

### The Present Study

Whereas the aforementioned studies already indicate that casual sexual scripts do occur in U.S. television shows, they do not provide any further information related to these casual sexual scripts. For instance, such findings raise the question whether and with what frequency such sexual encounters are repetitive and lead to a casual sexual relationship or remain casual non-repetitive sexual encounters. When a sexual act is shown between two individuals in a committed relationship, viewers receive a different message than when exposed to a casual sexual experience. Moreover, when studying such sexual portrayals, researchers also argue that a good understanding of “sexual behavior” in the media is essential. The large majority (80%) of sexual behavior in the media is generally restricted to physical flirting and romantic kissing (Bond, 2014). When focusing solely on sexual intercourse, Kunkel et al. (2005) found that only 10% of the 261 programs broadcasted in 2005 explicitly portrayed intercourse behavior. Such findings thus raise the question which sexual behaviors are typically shown within these casual sexual scripts.

RQ1: Is the type of sexual behavior shown in popular U.S. television programs related to the relational context of the sexual behavior?

Second, it is important to note that sexual content is not equally spread over all genres or channels of content but might be overrepresented in some and absent in others (Bilandzic & Busselle, 2012). Empirical studies showed that effects related to exposure to sexual content vary by genre (e.g., Gottfried, Vaala, Bleakley, Hennessy, & Jordan, 2013). Additionally, several content analyses demonstrated that some genres are more likely to show sexual portrayals compared to others (e.g., Bond & Drogos, 2014; Fisher et al., 2004; Kunkel et al., 2005, 2007). Fisher et al. (2004), for instance, found that comedy drama (i.e., a category that includes shows such as Sex and the City, Mind of the Married Man, and Ally McBeal) is the genre with the highest prevalence of sexual content and talk. When comparing comedy series, drama series, movies, news magazines, soap operas, talk shows, and reality shows, Kunkel et al. (2007) found that the comedy genre had the largest average number of scenes per hour containing sex, but the average level of sexual behavior in scenes was slightly higher for the drama genre compared to all other genres. Furthermore, these genres do not only differ in the frequency of sexual portrayals, but also in the context related to these sexual portrayals. Comedies, for instance, have significantly fewer risk and responsibility messages compared to shows that fall into the drama category (Gottfried et al., 2013). It could thus be that these genres also differ with regards to the relational context of the sexual behaviors.

RQ2: Is the relational context of the sexual behavior related to the genre of the popular U.S. television programs?

The next research questions are related to the casual sexual scripts. As mass media play an important role in conveying cultural scenarios (Wiederman, 2015), television, together with other media, plays a crucial role in influencing the cultural script, which in turn impacts the interpersonal and intrapsychic scripts. Regarding the casual sexual experience script in reality, alcohol is often cited as a contextual factor that facilitates engagement in casual sex (e.g., Littleton et al., 2009; Lyons et al., 2014). In the college environment, college students will often gather together in large groups, consume a decent amount of alcohol, and pair off as the evening progresses (e.g., Bogle, 2008; Wade, 2017). Race and class also seem to guide the casual sexual experience script, as studies found that mainly white and middle-class students report engagement in casual sex, whereas Hispanic, African American, and Asian American students report significantly fewer casual sexual experiences (e.g., Allison & Risman, 2014; Eaton et al., 2016; Wade, 2017) or even describe such casual sexual scripts as a traditional date (Littleton et al., 2009). Some argue that the cultural sexual script deployed by both mainstream and sexually explicit media is that casual sexual experiences are normative, fun, and recreational (e.g., Wright, 2009). Therefore, the third research question is formulated as follows:

RQ3: What is the casual sexual experience script in popular U.S. television shows?

Regarding the casual sexual relationship script, it is important to note that several types of casual sexual relationships exist. Mongeau et al. (2013), for instance, differentiated 7 types of casual sexual relationships based on the nature of the relationship and interactions between partners, including history of, or desire for, committed relationships. Desiring a romantic relationship is often a motive to engage in casual sex (e.g., Garcia & Reiber, 2008); others agree on a casual sexual relationship if that is “all they can get” because their romantic feelings are not mutual (Karlsen & Træen, 2013). In their descriptions of casual sexual scripts, women even described a man who led the woman to believe that he was interested in a long-term relationship when in fact he was only interested in sex (Littleton et al., 2009). Finally, as former partners often continue a casual sexual relationship after breaking up, a casual sexual relationship can be the result of a committed relationship once partners break off their commitment (Halpern-Meekin, Manning, Giordano, & Longmore, 2012). Given the numerous categories of casual sexual relationships, one might wonder which of these casual sexual relationships are often portrayed on the screen. Consequently, the final research question is formulated as follows:

RQ4: What is the casual sexual relationship script in popular U.S. television shows?

## Method

### Program and Episode Selection

For the purpose of this study, three genres that have been shown to repeatedly portray sexual behaviors (e.g., Fisher et al., 2004; Kunkel et al., 2007) and have a storyline that is strongly focused on relationship issues were selected (i.e., situational comedy, drama, and drama comedy). Given that fiction produced in the U.S. holds a dominant position in other countries (e.g., De Bens & De Smaele, 2001) and thus are often viewed outside the U.S. (e.g., Brown et al., 2013; Eyal, Raz, & Levi, 2014; Miller et al., 2016), we decided to analyze internationally popular U.S. television shows related to this topic seen its importance in terms of globalization and Americanization (Eyal et al., 2014).

Therefore, we chose three different programs produced in the U.S. within every genre. To select shows within those genres, programs aired between 2000^1^ and 2015 were chosen, given that the subject of casual sex has received quite some attention in academia since 2000 (e.g., Paul et al., 2000). For the purpose of this study, we aimed to include shows that are known for their portrayals of abundant and vivid sex scenes (i.e., *Californication* and *Girls*; Iftene, 2016) and were pioneers with regard to cultural changes related to sexuality (e.g., *Sex and the City, Orange Is the New Black*; Arthurs, 2003; Jensen & Jensen, 2007). In addition, we aimed to include U.S. produced television shows that have reached a worldwide popularity (e.g., *Grey’s Anatomy, The Big Bang Theory*; Adalian, 2015). We also wanted to include shows depicting emerging adults (e.g., *Gossip Girl*, *Girls*), as emerging adults are often subjects of studies examining casual sexual behaviors (e.g., Claxton & van Dulmen, 2013; Heldman & Wade, 2010). While this sample includes shows that are no longer in production, such as *Friends* and *Sex and the City*, it is important to note that these shows are still very popular, especially among international audiences (Brown et al., 2013; Sternbergh, 2016).

Since previous research noted that media effects are dependent on whether the program content is perceived as being realistic or not (Taylor, 2005), it was proposed that viewers may not strongly identify with situations that are not set within this world (e.g., *Game of Thrones*) or characters that possess supernatural powers (e.g., *Vampire Diaries*), despite the frequency of sexual content within such programs. In addition, series not set within the current time period were not included in our sample (e.g., *Downton Abbey, Vikings*). Popular movie and television shows databases (e.g., IMDb) and streaming services (e.g., Netflix) were consulted to ascertain whether (1) the television shows we selected were rated as popular by these databases and streaming services and (2) whether they could be viewed on other countries through streaming services such as Netflix or are still being broadcasted internationally (despite not being currently in production in the U.S.).

Regarding episode selection, every first and last episode of every season of every show was selected as recommended by Manganello, Franzini, and Jordan (2008). According to Manganello et al., sexual behaviors are most likely shown in the first and last episodes of the season, to create suspense and capture the viewers’ attention. Consequently, for the purpose of this study, we decided to act upon this recommendation. Additionally, one to four episodes, depending on the episode length and the number of seasons coded, were selected using a random number generator. When episode length was significantly longer compared to other programs and/or genres, not all seasons were analyzed (i.e., *Gossip Girl*,^2^ and *Grey’s Anatomy*) in order to have a comparable amount of total hours coded per genre. In total, 200 episodes were subjected to this content analysis, resulting in 102.65 coded hours of television content. Coders distinguished a total of 4301 scenes, of which 9.14% contained a form of sexual behavior (see Table 1 for more information on selected television programs and genres). A scene was defined as a collection of shots taken over one action or one event in the same location and at the same time. Coders were instructed to code a new scene when a time shift occurred (e.g., flashback of flashforward, dream, fantasy, etc.), a new event took place, and/or when the location changed clearly. Intercoder reliability will be discussed in the last part of this methods section.

**Table 1:** Information on selected television shows and genres in sample

Genre/show	Seasons (episodes) coded	Total hours coded	Total number of scenes	Scenes with sexual behavior	Sexual behavior within casual sexual experiences	Sexual behavior within casual sexual relationships
*Situational comedy*	16 (88)	32.94	1376	124 (9.01%)	23	15
Friends (2000–2004)	7–10 (24)	10.33 (620 min)	375	45 (12%)	3	8
The Big Bang Theory (2007–2015)	1–8 (40)	13.83 (829.5 min)	489	35 (7.16%)	11	1
New Girl (2011–2015)	1–4 (24)	8.78 (527 min)	512	44 (8.59%)	9	6
*Drama*	12 (48)	38.37	1787	94 (5.26%)	38	14
Grey’s Anatomy (2005–2009)	2–7 (18)	13.17 (790 min)	690	24 (3.48%)	11	3
Gossip Girl (2007–2012)	3–5 (18)	12.6 (756 min)	630	45 (7.14%)	20	6
Orange Is the New Black (2013–2015)	1–3 (12)	12.6 (756 min)	467	25 (5.35%)	7	5
*Comedy drama*	15 (64)	31.34	1138	175 (15.38%)	60	42
Sex and the City (2000–2004)	3–6 (20)	10.23 (614 min)	450	63 (14%)	11	21
Californication (2007–2014)	1–7 (28)	13.28 (797 min)	406	83 (20.44%)	39	13
Girls (2012–2015)	1–4 (16)	7.83 (470 min)	282	29 (10.28%)	10	8
Total	43 (200)	102.65	4301	393	121	71

### Unit of Analysis

The unit of analysis for this study was a sexual behavior coded at the scene-level, which was divided into three different categories, being (1) passionately kissing and intimate touching, (2) explicit oral/vaginal/anal sex, and (3) implied oral/vaginal/anal sex. The coding scheme identified four different contexts in which such sexual behaviors could occur: (1) *casual sexual experiences*, (2) *casual sexual relationships*, (3) *dates*, and (4) *committed relationships*.

Coders identified a casual sexual experience based on the definition by Garcia and Reiber (2008, p. 193), in which it is described as “a spontaneous sexual interaction in which: (1) the individuals are explicitly not in a traditional romantic relationship with each other (i.e., not dating, not boyfriend/girlfriend), (2) there are no a priori agreements regarding what behaviors will occur, and (3) there is explicitly no promise of any subsequent intimate relations or relationships.” Furthermore, coders provided more information on characteristics of the casual sexual experience (i.e., sex of the initiator, prior relationship between casual sex partners, outcome^3^ of the casual sexual experience, any form of aggression during the casual sexual experience, any explicit or implicit use of contraception during the sexual act, and alcohol or drug influence), demographic information of the characters that performed the sexual behavior (i.e., sex, age, ethnicity, sexual orientation, relationship status), and characters’ displays of permissiveness (i.e., character is portrayed as someone who enjoys sex without love, character avoids commitment, and character cheats on partner). Coders were encouraged to code displays of permissiveness on the episode—or season-level, as such coding is often not clear based on the scene-level. A character was coded as someone who enjoys sex without love when that character, for instance, did not show feelings of regret after a casual sexual experience. Examples for coding characters as avoiding commitment include portrayals in which the character talks to other characters about commitment issues or portrayals of when the character is shown to be afraid of losing independence.

For casual sexual relationships, the type of casual sexual relationships was coded, following an existing typology on casual sexual relationships, which simultaneously served as definitions of casual sexual relationships (Mongeau et al., 2013). The first category, *true friends,* reflects the typical friends with benefits relationship in which close friends interact in varied contexts and have sex on multiple occasions. The second category, *just sex,* reflects the typical fuck buddy relationship, in which casual sexual partners interact almost exclusively to arrange and carry out sexual interaction. The third category, *network opportunism*, refers to friends or acquaintances who share network ties and engage in casual sex whenever convenient. The next three categories are related to the desire for a committed relationship being *successful transition in a committed relationship*, *unintentional transition in a committed relationship*, and *failed transition in a committed relationship*. The final category is *transition out of a committed relationship* (Mongeau et al., 2013). As the type of casual sexual relationship is often not clear based on the scene-level or in some cases even at the episode-level, coders were instructed to code the casual sexual relationship at the season-level. Coders also provided more information on characteristics of the casual sexual relationship (i.e., any form of aggression during the sexual behavior, any explicit or implicit use of contraception), demographics of characters that engaged in casual sexual relationships (i.e., sex, age, ethnicity, sexual orientation, relationships status) as well as characters’ displays of permissiveness (i.e., character is portrayed as someone who enjoys sex without love, character avoids commitment, and character cheats on partner).

Before any coding occurred, all main characters across the nine television shows were listed in the variable “main character”, which resulted in a variable that consisted of 108 values. When the character was not part of the characters listed in this variable, it was coded as a “secondary character” and coders also provided the name of this secondary character.

### Intercoder Reliability

Twenty-five undergraduate and two graduate students underwent extensive training to learn how to implement the coding system developed by the first author, which included measurement of variables and coding rules made before the observations as recommended by Neuendorf (2002). Undergraduate students served as coders blind to the purpose of the original study. Pilot coding occurred on several episodes before coding the actual sample to identify and resolve problems with the coding scheme. As coders had difficulties in separating scenes, a word sheet was designed in which coders separated scenes and indicated which scenes needed further coding. This procedure resulted in more focus while coding, increased accurate separating of scenes and facilitated feedback, which was regularly provided during the training phase to all coders by the first author. After the coding scheme was modified on the basis of these practice rounds and once coders reached consensus on separating scenes, the coding of the episodes was independent and coders started coding episodes belonging to the dataset of this study.

Using a random number generator, 43 episodes from the original 200 episodes were randomly selected and subjected to reliability analyses. Subsequently, 21.5% of the sample was coded by two coders, as recommended by Neuendorf (2002). Intercoder reliability was computed for this subsample and was measured through Krippendorff’s alpha (kalpha) coefficient (Hayes & Krippendorff, 2007). Although it was previously stated that kalpha should be around .80 (Neuendorf, 2002), Hayes and Krippendorff (2007, p. 87) argue that “if the reliability standard were relaxed to *α*_min_ = .700, the risk of accepting the data as reliable when they are not is quite low, *q* = .0125.” Consequently, we decided to delete all variables with kalpha’s lower than .70 from all analyses.

Overall, the coders agreed well on the unitizing of the episodes into scenes (*α* = .998). Reliabilities for the variables on the scene-level were: context of sexual behavior (*α* = .81) and type of sexual behavior (*α* = .84). Reliabilities for variables further coded on the casual sexual experience-level were: sex initiator of the casual sexual experience (*α* = .83), prior relationship between casual sex partners (*α* = .90), outcome of the casual sexual experience (*α* = 1.00), alcohol or drug influence (*α* = .78), any explicit or implicit use of contraception during the sexual act (*α* = 1.00), demographic variables such as character’s sex (*α* = .97), character’s age (*α* = .79), ethnicity (*α* = 1.00), sexual orientation (*α* = .79), relationship status (*α* = .85), and the character’s enjoyment of sex without love (*α* = .82). The following variables were deleted from all analyses due to low kalpha values: any form of aggression during the casual sexual experience (*α* = .67), the character is portrayed as someone who avoids commitment (*α* = .40), and whether the character cheats on partner because of the casual sexual experience (*α* = .69).

Reliabilities for variables on the casual sexual relationship-level were: type of casual sexual relationship (*α* = .89), any form of aggression during the sexual behavior (*α* = 1.00), any explicit or implicit use of contraceptives during the sexual behavior (*α* = .99), demographic variables such as character’s sex (*α* = 1.00), age (*α* = 1.00), ethnicity (*α* = 1.00), sexual orientation (*α* = 1.00), relationship status (*α* = .70), the character’s enjoyment of sex without love (*α* = .70), the character’s portrayal as someone who avoids commitment (*α* = 1.00), and whether the character cheats on partner because of the casual sexual relationship (*α* = 1.00). No variables were deleted from analyses related to the casual sexual relationship script, as there were no unreliable kalpha values.

## Results

### RQ1: Is the Type of Sexual Behavior Shown in Popular U.S. Television Programs Related to the Relational Context of the Sexual Behavior?

In total, 393 scenes (9.14% of all scenes) were coded that portrayed some act of sexual behavior. When examining the context of those sexual behaviors, the results showed that almost one-third (*n* = 121; 31%) of these sexual behaviors occurred within a casual sexual experience context. Additionally, 18% (*n* = 71) of these sexual behaviors happened within a casual sexual relationship. In contrast, half of those sexual behaviors occurred within a committed relationship or date (*n* = 201; 45% committed relationship; 6% date).

When taking into account the type of sexual behavior, a chi-square test showed that the large majority (*n* = 152; 76%) of the sexual intimacies within a romantic relationship concern portrayals of passionate kissing and intimate touching, whereas 77% (*n* = 56) of explicitly portrayed oral, vaginal, or anal sexual intercourse occurred within a casual sexual experience (*n* = 33; 45%) or a casual sexual relationship (*n* = 23; 32%) context. When it comes to implied sexual behavior, romantic relationships (*n* = 32; 42%) and casual sexual experiences (*n* = 31; 40%) barely differed, whereas this number was notably smaller for casual sexual relationships (*n* = 14; 18%). This association between the type of sexual behavior and the context of sexual behavior appeared to be significant, *χ*^2^(4) = 39.58; *p* < .001. For portrayals of passionate kissing and intimate touching, the standardized residual was significant for casual sexual experiences (*z* = − 2.1) and romantic relationships/dates (*z* = 2.5), implying that significantly more passionate kissing and intimate touching occurred within a romantic relationship/date, while significantly less passionate kissing and intimate touching occurred within casual sexual experiences. In contrast, the standardized residual for explicit portrayals of oral, vaginal, or anal sex was significant for casual sexual experiences (*z* = 2.2), casual sexual relationships (*z* = 2.7), and committed relationships/dates (*z* = − 3.3), signifying that such behaviors were significantly more likely to occur within casual sexual experiences and relationships, and less likely to occur within committed relationships/dates. No significant differences were found for implied sexual behavior (see Table 2).

**Table 2:** Association between the type of sexual behavior, the context of sexual behavior, and the three genres

Genre	Type of sexual behavior	Context sexual behavior			Total
		Casual sexual experiences	Casual sexual relationship	Committed relationship	
Situational comedy	Kissing and touching	13	11	69	93 (75%)
	Explicit sex	0	0	0	0
	Implied sex	10	4	17	31 (25%)
	Total (%)	23 (19%)	15 (12%)	86 (69%)	124
Drama	Kissing and touching	22	7	36	65 (69%)
	Explicit sex	6	3	0	9 (10%)
	Implied sex	10	4	6	20 (21%)
	Total (%)	38 (40%)	14 (15%)	42 (45%)	94
Comedy drama	Kissing and touching	22	16	47	85 (49%)
	Explicit sex	27	20	17	64 (37%)
	Implied sex	11	6	9	26 (15%)
	Total (%)	60 (34%)	42 (24%)	73 (42%)	175
Total	Kissing and Touching	57	34	152	243
	Explicit sex	33	23	17	73
	Implied sex	31	14	32	77
	Total	121 (31%)	71 (18%)	201 (51%)	393

### RQ2: Is the Relational Context of the Sexual Behavior Related to the Genre of the Television Program?

Casual sexual experiences were proportionately most often portrayed in the drama genre (*n* = 38; 40%), followed by the comedy drama genre (*n* = 60; 34%), whereas only 19% (*n* = 23) of portrayals of sexual behaviors in situational comedy occurred within a casual sexual experience context. A chi-square test confirmed that casual sexual experiences (*z* = − 2.5) appeared less often in the situational comedy genre compared to the other genres, *χ*^2^(4) = 27.79; *p* < .001. Regarding casual sexual relationships, sexual behaviors within this context most often occurred in the comedy drama genre (*n* = 42; 24%), whereas they were not that often portrayed in the drama genre (*n* = 14; 15%) nor in the situational comedy genre (*n* = 15; 12%). Yet, these differences were not significant.

Finally, sexual behaviors were most often showed within a committed relationship for situational comedies (*n* = 86; 69%), whereas these frequencies were a bit lower for the drama genre (*n* = 42; 45%) and the comedy drama genre (*n* = 73; 42%). The significant chi-square test (*χ*^2^[4] = 27.79; *p* < .001) indicated that sexual behaviors within a committed relationship (*z* = 2.8) appeared more often in the situational comedy genre compared to the other genres (see Table 2).

### RQ3: What is the Casual Sexual Experience Script in Popular Television Shows?

**Fig. 1:**
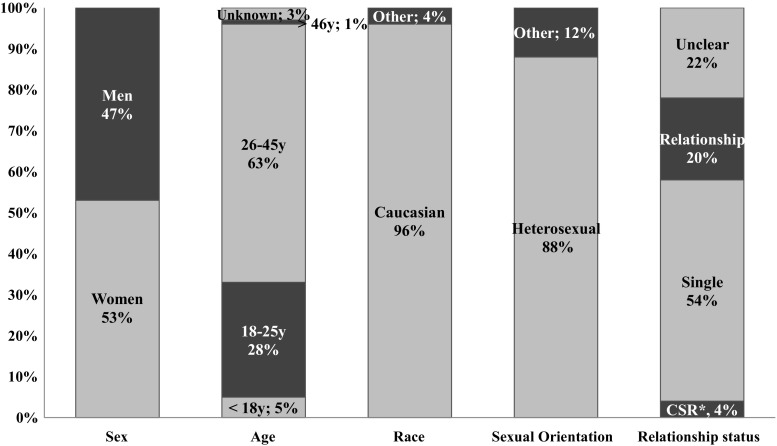
Characters’ demographics in the casual sexual experience script. *CSR casual sexual relationship

Across the nine television programs, 121 casual sexual experience cases were analyzed. First, the character’s demographics were examined as illustrated in Fig. 1. Slightly more females (53%) than males (47%) engaged in hooking up behavior. Most casual sex partners (63%) were in the adults age category (26–45-year-olds), followed by the emerging adulthood category (18–25-year-olds, 28%). Only a small minority of casual sex partners were teenagers (5%) or older adults (46–65-year-olds; 1%). Almost all characters that had a casual sexual experience were Caucasian (96%) and heterosexual (88%). For half of characters, it was clear they were single while having the casual sexual experience (54%). Contrarily, 49 characters (20%) were in a committed relationship while having a casual sexual experience and thus cheated on their significant other, and 9 characters (4%) were involved in a casual sexual relationship. For 54 characters (22%), the relationship status was unclear based on the episode. Most characters that had a casual sexual experience were portrayed as enjoying sex without love (56%). No significant differences were found for both male and female characters regarding cheating on their partners; *χ*^2^(2) = .87, *p* = .65. Contrarily, less male characters (13%) did “not enjoy having sex without love” (*z* = − 2.1), *χ*^2^(1) = 11.43, *p* < .01. This was not the case for female characters (51%) and male (62%) characters that enjoyed sex without love and females that did not (34%).

**Fig. 2:**
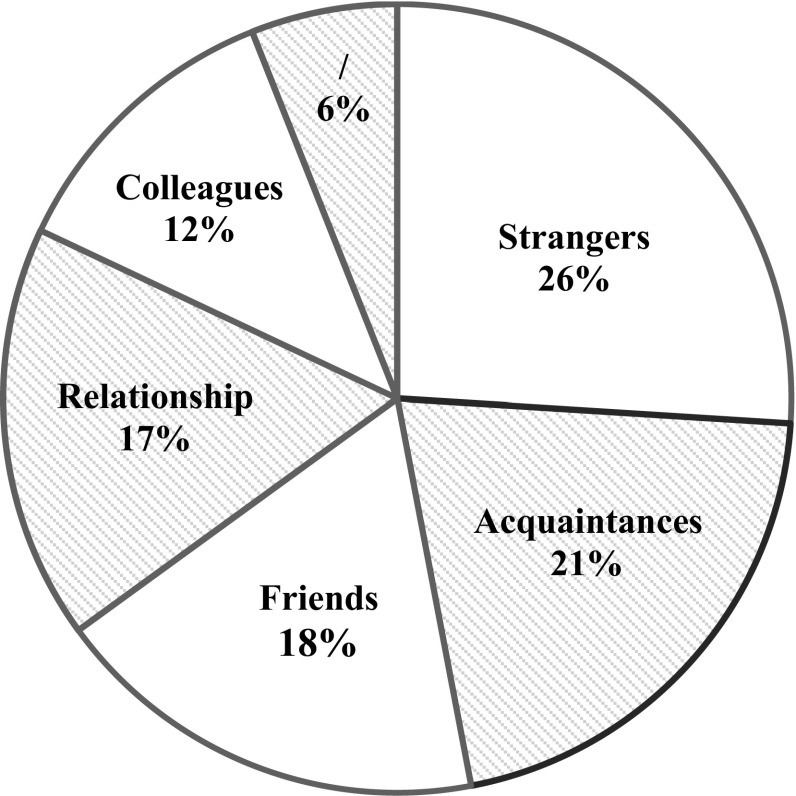
Prior relationship between casual sexual partners in the casual sexual experience script

**Fig. 3:**
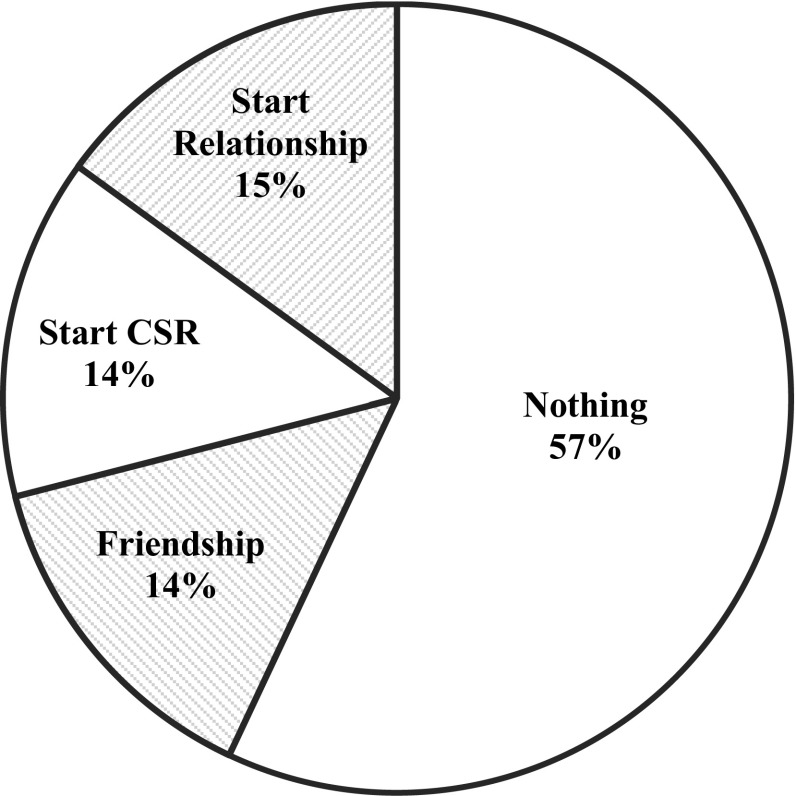
Outcome of the casual sexual experience in the casual sexual experience script

Next, we looked at the relationship between the casual sex partners prior to their casual sexual experience (see Fig. 2). In 32 cases (26%), the casual sex partners were strangers. In 25 cases (21%), they were acquainted, in 22 cases (18%) they were friends, and in 20 cases (17%) they were previously romantically involved. In the smallest category of cases (12%), they were colleagues or neighbors. For 8 cases, the coders indicated the prior relationship was unknown based on the episode. Secondly, we examined the outcome of the casual sexual experience, which is illustrated in Fig. 3. As expected, in 69 cases (57%) the casual sexual experience did not lead to anything. However, in 17 cases (14%) the casual sex partners became friends, in another 17 cases (14%) they commenced a casual sexual relationship, and in 18 cases (15%) they even established a committed relationship. When paying attention to which character typically initiated the casual sexual encounter, female characters (35%) were more likely to initiate the casual sexual encounter compared to male characters (25%). In 19 cases (16%), both characters initiated the casual sexual encounter, and in 31 cases (26%) it was not clear which character initiated it. Only in 2% of the sexual behaviors within the casual sexual experience context, the characters explicitly used or implicitly referred to any forms of contraception. In 18% of the casual sexual experience cases, at least one character was under influence of drugs or alcohol, indicating that being under influence does not necessarily facilitate casual sexual experiences on the screen.

### RQ4: What is the Casual Sexual Relationship Script in Popular Television Shows?

**Fig. 4:**
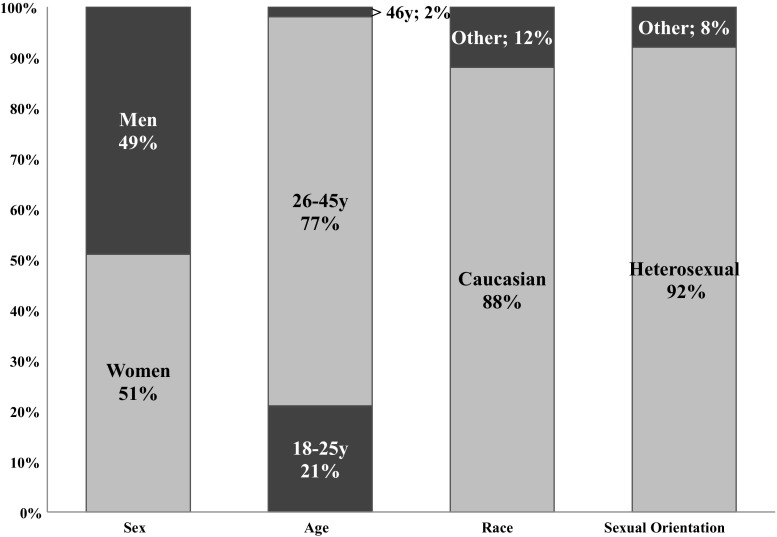
Characters’ demographics in the casual sexual relationship script

In total, 71 sexual behaviors occurred within a casual sexual relationships in the nine television programs. Given that casual sexual relationships occur between two people and almost all characters (92%) were heterosexual, male characters (49%) and female characters (51%) did not differ regarding their engagement in casual sexual relationships. Interestingly, characters engaging in a casual sexual relationship were mostly main characters (71%), compared to secondary characters (29%). In line with the casual sexual experience script, a large majority of characters were Caucasian (88%) and 26–45-year-olds (77%). Approximately one-fifth of characters (21%) were emerging adults (18–25-year-olds) and only two characters were older than 45 (see Fig. 4). Two-third of characters (66%) were portrayed as someone who enjoys sex without love, but solely 25% avoided commitment. Only 3 characters cheated on their significant other because of the casual sexual relationship. Interestingly, men and women did not differ when it comes to enjoying sex without love (*χ*^2^[1] = .37, *p* = .587) or avoiding commitment (*χ*^2^[1]= .51, *p* = .551).

**Fig. 5:**
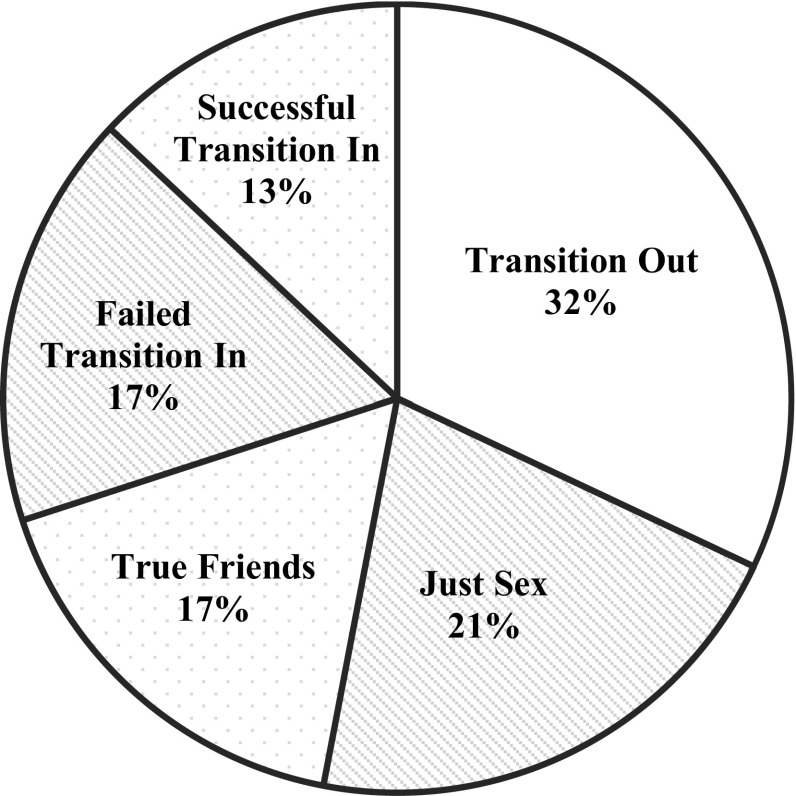
Type of casual sexual relationship

As illustrated in Fig. 5, the casual sexual relationship was in most of the cases (*n* = 23; 32%) a result of two characters that transitioned out of a committed relationship, also commonly referred to as “ex-sex.” In 15 cases (21%), the casual sexual relationship was restricted to just sexual activities, whereas 12 cases (17%) portrayed a typical friend with benefits relationship in which the characters were true friends and did not expect any romantic relationship out of the casual sexual relationship. Contrarily, in 21 cases (30%), at least one of the partners was hoping the casual sexual relationship would evolve into a romantic relationship. Yet, this transition was successful in only 9 cases, resulting in 12 casual sexual relationships classified as failed transition in a committed relationship (see Fig. 5).

## Discussion

Several researchers have expressed their concern regarding the amount and type of sexual portrayals in television content (e.g., Garcia et al., 2012; Heldman & Wade, 2010). Notwithstanding, sexual intercourse within a committed relationship is generally perceived as a normative and even desired behavior (Hicks, McNulty, Meltzer, & Olson, 2016), indicating that such concerns are relatively superfluous as long as sexual behaviors occur within a committed relationship. The results of this content analysis suggest that casual sexual experiences and relationships are almost as frequently shown in popular television programs as sexual behaviors within more traditional committed relationships. This is in line with previous findings in content analyses related to prior relationship or relationship status (e.g., Fisher et al., 2004; Kunkel et al., 2007). However, when taking into account the type of sexual behavior, casual sexual experiences and relationships consist mostly of explicit portrayals of sexual intercourse, whereas sexual behaviors within a committed relationship or date are mainly limited to passionate kissing. Such portrayals might give viewers the impression that casual sex has become the normative sexual script. In reality, however, sex in the context of a relationship is more likely to occur than sex in the context of a casual sexual experience (Fielder, Carey, & Carey, 2013).

Furthermore, genre seems to play an important role when it comes to examining sexual behavior within its context. The situational comedy genre, for example, had no explicit portrayals of intercourse and in less than one-third of the cases implied sex or kissing occurred within a casual sexual experience or relationship context. Notably, the situational comedy had the largest number of sexual behaviors that occurred within a romantic relationship compared to drama and comedy drama. However, the majority of these sexual behaviors were limited to kissing. Comedy drama, on the contrary, had the largest proportion of sexual behaviors within casual sexual relationships and the largest proportion of explicit sexual portrayals. Finally, the drama genre had the largest proportion of casual sexual experiences. Interestingly, situational comedy had more cases of implied sex compared to drama and comedy drama. Again, more than half of those portrayals of implied sex occurred within a romantic relationship for situational comedy, whereas the opposite was true for the drama genres and comedy drama. Such findings thus stress the importance of genre when studying attitudes or behavior related to exposure to sexual television content. Whereas the situational comedy genre might not be that detrimental when it comes to creating a casual sexual experience script or casual sexual relationship script, drama—and comedy drama in particular—might have a stronger influence on its viewers due to their promotion of casual sex.

For the third research question, the casual sexual experience script was analyzed across the three genres. Initially, casual sex partners were defined as strangers who do not hold any expectations toward relational outcomes (Garcia & Reiber, 2008). Yet, according to this content analysis, only in less than one-third of casual sexual experience cases, casual sex partners were strangers. Similarly, while previous research emphasizes the pervasiveness of alcohol use within casual sexual experiences (e.g., England et al., 2008; Wade, 2017), only in 18% of the cases at least one casual sex partner was under the influence of alcohol and drugs. The use of contraception barely occurred in the analyzed scenes portraying a sexual behavior within the casual sexual experience context. This is in line with findings from a content analyses by Kunkel et al. (2007), who concluded that topics related to sexual risks and responsibilities remain infrequent overall. It thus seems that findings related to the casual sexual experience script are not entirely congruent with earlier findings related to the casual sexual script in reality. Yet, when taking a look at fairly recent studies related to casual sex, some changes have occurred as well. For instance, while it still is more normative for men to initiate casual sex than it is for women, both genders believe it shouldn’t be this way (Uecker & Martinez, 2017). Remarkably, female characters in this content analysis were even more likely to initiate casual sex compared to male characters. It thus could be that when repeatedly exposed to the televised casual sexual experience script, viewers will eventually adapt such scripts in real life.

Additionally, in 43% of the casual sexual experience cases, the casual sex partners remained in contact, thereby evolving their relationship into either a romantic relationship, casual sexual relationship or friendship. Developing a friendship (e.g., Eaton et al., 2016) or wanting a committed relationship (Bradshaw et al., 2010) are often classified as motives and/or risks for hooking up. Similarly in the casual sexual relationship script, in 30% of the analyzed cases, at least one of the two characters involved was hoping the casual sexual relationship would evolve into a committed relationship. In reality, however, few casual sexual relationships actually lead to a committed relationship. For example, a study on friends with benefits relationships showed that on average, only 10% of respondents eventually became romantically involved with their casual sex partner (Bisson & Levine, 2009). When frequently exposed to these casual sexual scripts on the screen, individuals might, as Wade (2017) suggests, indeed perceive casual sex as a way to eventually obtain a committed relationship.

Moreover, the heterosexual script, in which women are more likely to seek commitment whereas men try to avoid it (e.g., Kim et al., 2007), does not seem the case for the televised casual sexual experience script, as female characters are also enjoying recreational sex on the screen. These findings are in line with a qualitative content analysis on the comedy drama series *Sex and the City* (Markle, 2008). Female characters in the televised casual sexual experience script were also more likely to initiate a casual sexual encounter compared to their male characters. Although this content analysis did not include consequences of the casual sexual scripts, several researchers argue that hooking up has negative psychological consequences in real life, especially for women (e.g., Bogle, 2008; Campbell, 2008; Fielder & Carey, 2010; Fisher, Worth, Garcia, & Meredith, 2012; Grello, Welsh, & Harper, 2006). Contrarily, research on casual sexual relationships suggests engagement in casual sexual relationships does not hold the same outcomes, as for both men and women the magnitude of positive emotional reactions about casual sexual relationships clearly surpassed the negative emotional reactions (Owen & Fincham, 2011). Moreover, not all studies found significant associations between casual sexual behavior and well-being (e.g., Eisenberg et al., 2009; Vrangalova, 2015b) or instead found positive associations with well-being (e.g., Vrangalova, 2015a; Vrangalova & Ong, 2014).

In her study on short-term longitudinal associations between several definitions of casual sex and indicators of psychological well-being, Vrangalova (2015a) showed that statistical associations between psychological well-being and casual sex are infrequent, with the majority of them leading to higher instead of lower well-being. Surprisingly, even, she found that women experienced higher and men experienced lower well-being after hooking up. It could be that, as women are repeatedly exposed to casual sexual scripts on the screen in which they witness that women can enjoy recreational sex as well, their intrapsychic scripts have gradually changed over time and due to this change they will experience less negative emotions related to their casual sexual experiences.

Regarding the casual sexual relationship script (RQ4), the results showed that sexual behaviors most frequently occurred between former partners, indicating that sex with a former partner is often shown on the screen. Casual sexual relationships offer former partners the possibility to continue sexual interactions even after breaking up (Mongeau et al., 2013), a behavior that is not that uncommon, as half of emerging adults who break up continue sexual interactions with their former partners (Halpern-Meekin et al., 2012). Remarkably, sexual behavior within casual sexual relationships most often occurred within the comedy drama genre. Characters engaging in such casual relationships were predominantly main characters, suggesting that such on/off-again relationships occur between main characters over seasons as being part of the storyline. Indeed, Hank and Karen (i.e., *Californication*), Carrie and Big (i.e., *Sex and the City*), and Hannah and Adam (i.e., Girls) were couples in the analyzed series that often break up but continued to have sexual interactions. This way, casual sexual relationships do not only replace committed relationships but also serve as a transition between the exclusivity of a romantic relationship and a total termination of the relationship. Yet, at the end of the series, these characters usually end up together (e.g., Carrie and Big in *Sex and the City*; Markle, 2008), which thus might create romantic beliefs when it comes to the engagement in casual sexual relationships, in which casual sex partners come to believe that they are destined to be together. Consequently, they might remain close to each other instead of moving on to a new relationship. However, future research is warranted to point out whether television creates unrealistic expectations toward casual sexual relationships.

### Limitations and Directions for Future Research

While this study provides interesting insights in casual sexual scripts, it is not without limitations. First, as we only included three television programs per genre, it is not recommended to generalize our findings to other programs within the same genre. While this content analysis discovered an interesting trend across these three television programs per genre, more content analyses are necessary to examine the reliability and generalizability of this trend. Moreover, our sample might have been a bit biased due to our sample selection. Given that we aimed to include television shows that have a strong focus on sexual portrayals (e.g., *Californication*, *Girls*, and *Sex and the City*), the amount and type of sexual portrayals within the comedy drama genre might be a bit overrepresented. However, it is important to note that a previous content analysis also reported that the comedy drama genre has the highest prevalence of sexual content and talk (Fisher et al., 2004). Yet, we encourage future content analyses related to casual sexual scripts to apply a systematic approach (e.g., randomly select shows within a genre or code U.S. prime time hours) when selecting their sample to further validate the findings of this study.

Based on our findings regarding the televised casual sexual scripts, the field seems to need additional research on casual sexual experiences and relationships that is not solely focused on college students, but also includes (older) adults. Although we tried to include series that focused on characters in emerging adulthood (i.e., *Gossip Girl* and *Girls*), the majority of series featured characters in their late twenties and thirties, or even forties. In a more representative sample of television shows, researchers also found that most characters involved in portrayals of sexual behavior were aged 25 or older (e.g., Kunkel et al., 2007). According to Schwartz (2010) Americans still hold a deep-rooted ambivalence about teens and young adults desiring sexual pleasure outside the bounds of intimate relationships, which could explain why there are less portrayals of those younger than 25 engaging in casual sex. However, it is important to note that our sample included several television programs with main characters in between their thirties and fifties (e.g., *Sex and the City, Californication*). It might thus be that series targeted at a younger age group (e.g., those broadcasted on Disney Channels) or reality dating series which often include participants between the ages of 18 and 30 (e.g., *Are You the One? * and *Ex On the Beach*) portray a different casual sexual experience or casual sexual relationships script.

Finally, it is important to note that a content analysis is merely an attempt to form an idea about the televised casual sexual experience script and does not allow us to make any predictions about effects on those exposed to the televised casual sexual scripts. Nonetheless, this content analysis provides a basis for identifying messages to be examined in experiments and quantitative surveys (Slater, 2013). Therefore, additional studies applying these research methods are needed to gain a better understanding of the impact of these cultural messages concerning these casual sexual scripts that are worldwide disseminated through popular television shows originated in the U.S.

## Footnotes

1 In case the show started before 2000, we only coded seasons that started after 2000. This was the case for *Friends* and *Sex and the City*.

2 In the case of *Gossip Girl* we decided to only code those episodes in which the main characters have reached emerging adulthood.

3 The casual sexual experience outcome could not be coded on the scene-level. Coders were encouraged to code the casual sexual experience outcome on the season-level and verify their decision by collecting more information on the characters through internet sources.
